# How a Man Walks

**Published:** 1883-09

**Authors:** 


### HOW A MAN WALKS.

One of the most remarkable things about a man's walk, says *Science for All*, is the diagonal movement which characterizes it. The hands and feet may be regarded as forming the four corners of a parallelogram, and the diagonal limbs are, of course, the right arm and left leg and the left arm and right leg. By "diagonal movement" is meant that the diagonal limbs during locomotion always swing in the same direction. The arms swing by the body like a couple of pendulums, and with a speed which entirely depends upon the rate at which he may be walking. The athlete, anxious to complete the given number of "laps" in a mile or couple of miles and outstrip his competitor, swings his arms to and fro with a quickness which corresponds with the motion of his swift feet; the business man also swings his arms with a motion which, if not so quick, exactly times with the motion of his legs. Now, if the motion be even carelessly observed, it will be found that the right arm swings forward at the same time as the left leg, and when the right leg is advancing it is the left arm which accompanies it. This diagonal movement of the limbs is the natural method adopted by man when walking, and it is the first and most apparent fact that one ascertains in studying human locomotion.

